# A novel *MFSD8* mutation in a Russian patient with neuronal ceroid lipofuscinosis type 7: a case report

**DOI:** 10.1186/s12881-018-0669-7

**Published:** 2018-08-25

**Authors:** Anastasiya Aleksandrovna Kozina, Elena Grigorievna Okuneva, Natalia Vladimirovna Baryshnikova, Anna Yurievna Krasnenko, Kirill Yurievich Tsukanov, Olesya Igorevna Klimchuk, Olga Borisovna Kondakova, Anna Nikolaevna Larionova, Tatyana Timofeevna Batysheva, Ekaterina Ivanovna Surkova, Peter Alekseevich Shatalov, Valery Vladimirovich Ilinsky

**Affiliations:** 1 Institute of Biomedical Chemistry, Pogodinskaya street 10 building 8, 119121 Moscow, Russia; 2 Genotek Ltd, Nastavnicheskii pereulok 17/1, 105120 Moscow, Russia; 3 Pirogov Russian National Research Medical University, Ostrovitianova street 1, 117997 Moscow, Russia; 4 Scientific and Practical Centre of Pediatric psychoneurology of Moscow Healthcare Department, Michurinsky prospect, 74, 119602 Moscow, Russia; 5 Veltischev Research and Clinical Institute for Pediatrics of the Pirogov Russian National Research Medical University, Taldomskaya str 2, 125412 Moscow, Russia; 6 Vavilov Institute of General Genetics, Gubkina street 3, 119333 Moscow, Russia

**Keywords:** Neuronal ceroid lipofuscinosis, NCL, *MFSD8*, NCL7, Variant late-infantile NCL, Rett-like phenotype

## Abstract

**Background:**

Neuronal ceroid lipofuscinoses (NCLs) are the most common autosomal recessive neurodegenerative disorders in children. Clinical manifestations include progressive cognitive decline, motor impairment, ataxia, visual loss, seizures and early death. To date more than 440 NCL-causing mutations in 13 genes are known.

**Case presentation:**

We report clinical and genetic characteristics of a 5-year-old girl affected by ceroid lipofuscinosis type 7 (NCL7). She had progressive motor and mental deterioration since the age of 2,5 years. Later she developed progressive vision loss, stereotypies, action myoclonus and epilepsy. By the age of 5 years she stopped walking. Based on symptoms, diagnosis of Rett syndrome was suggested, but no abnormalities were detected in *MeCP2.* We identified a novel homozygous mutation in *MFSD8* gene (c.525 T > A, p.Cys175Ter). To our knowledge, this is the first report of *MFSD8* gene mutation in a Russian patient with variant late-infantile NCL.

**Conclusions:**

Our results enlarge mutational spectrum of ceroid lipofuscinosis type 7 and demonstrate tremendous diagnosis value of exome sequencing for pediatric NCLs. Also we confirmed that NCL should be suspected in patients with Rett-like phenotype at onset and negative *MECP2* mutation.

## Background

Neuronal ceroid lipofuscinoses (NCLs), also known as Batten disease, are a group of autosomal recessive lysosomal storage diseases. Autosomal dominant inheritance has been reported in one adult-onset form [1]. NCL is the most common of neurodegenerative disorders of childhood with prevalence up to 1:14,000 worldwide [2]. NCLs are associated with progressive loss of cognitive and motor skills, seizures, myoclonus, loss of vision, and usually reduced life expectancy. The age of onset can be variable. Almost all NCL patients had accumulation of autofluorescent lipopigment in lysosomes of neurons and other cell types. This storage process is associated with selective destruction and loss of neurons in brain and retina. The ultrasructure of the storage deposits varies between different forms of NCL [3].

Previously, NCL classification was based on age of onset together with clinical presentation. Patients were grouped in one of four basic NCL types: infantile, late infantile, juvenile and adult [4].

To date more than 440 NCL-causing mutations in 13 genes are known [5]. The new classification structured in 7 diagnostic axes: responsible gene, precise genetic defect, clinical characteristics (age at onset, presenting symptoms, disease progression), biochemical phenotype, ultrastructural features, functionality and other remarks [6]. But a direct correlation between the gene that is mutated and phenotype does not always exist [7].

Within late infantile NCLs, several types with discreet different clinical characteristics are described and separated into variant late infantile NCL (vLINCL). vLINCLs are genetically heterogeneous forms with four major disease-causing genes: *CLN5*, *CLN6*, *CLN7* (*MFSD8*), *CLN8*. Homozygous or compound heterozygous mutations in *MFSD8* were previously reported to cause vLINCL called NCL7 disease (OMIM 610951). *MFSD8* gene (OMIM 611124) encodes CLN7, a putative lysosomal transporter protein [8].

NCL7 form was first described in children from Turkey: Topcu with colleagues evaluated clinical and histopathologic features of 36 Turkish patients with late-infantile NCL [9]. This form was considered a distinct clinical and genetic variant of NCL, but later studies showed that NCL7 disease is not limited to Turkish population [8, 10, 11]. It is now evident that Turkish vLINCL is genetically very heterogeneous with mutation in three genes: *CLN6* [12], *CLN8* [13] and *MFSD8* [14]. Clinical phenotype of patients with different variants of infantile and late infantile NCLs is quite uniform. However, Rett-like onset have been described for NCL7 disease, produced by *MFSD8* gene mutations, and infantile NCL1 disease [9, 15, 16]. Similar autistic characteristics and stereotypic movements were observed in several forms of NCL [17, 18].

In this study we analyzed clinical and genetic characteristics of a 5-year-old girl with cognitive and motor deterioration, vision loss, stereotypies, action myoclonus and epilepsy.

## Case presentation

The patient was a 5 year-old girl from Russia. She had unremarkable perinatal, neonatal and family history (parents and brother are clinically healthy).

She was born from the fifth pregnancy, the second childbirth and was delivered by Caesarean section. Her birth weight was 3800 g and height was 53 cm. Apgar scores were 8 and 8 at 1 and 5 min respectively. No abnormalities were noted in neonatal period. Up to 2.5 years the girl developed according to her age without delay of speech and motor development. At the age of 2,5 years against a background of trauma of little finger, girl stopped talking. Gradually speech was restored, but vocabulary decreased. At 3 years the first febrile seizure attack occurred. Later parents noticed significant deterioration in her speech and communication. She became socially withdrawn. Brain magnetic resonance imaging revealed diffuse lesions in the white matter and hypoplasia of the lower cerebellar vermis. At the age of 3, 5 years stereotypic movements appeared. From 3, 5 years patient was commenced on valproic acid (antiepileptic drug). But motor deterioration progressed: by the age of 5 she stopped walking.

Based on observed symptoms, diagnosis of Rett syndrome was suggested. Prior to clinical exome sequencing the following studies were carried out: measurement of palmitoyl protein thioesterase (PPT) level in leukocyte, tandem mass spectroscopy, sequencing of *MeCP2* and *TPP1*, analysis of common mitochondrial DNA mutations. All studies showed no abnormalities.

At the age of 5 years 8 months she was admitted to Scientific and Practical Center of Pediatric Psychoneurology with motor and mental deterioration, visual impairment and stereotypies.

She had normal physical development: she was 20, 5 kg in weight and 111 cm in height. Head was normal shape, head circumference was 50, 5 cm (normal). The skin was normal and clean. Abdomen was soft, painless. Stool and micturition were normal. Basic blood and urine tests were normal.

There was no interest in environment, no play activity. Orientation in space and time was absent. Speech and understanding of speech is disturbed: she used only speech sounds and syllables. She had stereotypic movements of hands and face. The girl have myoclonus in her hands, legs and facial muscles. Tactile stimulation enhances myoclonus. She does not walk, does not stand, does not crawl. A girl can only hold her head, roll over, sit with periodic falls.

Ophthalmological evaluation revealed partial atrophy of optic nerves, nistagmus, retinitis pigmentosa and mixed astigmatism.

EEG (electroencephalography) revealed a significant delay in the formation of cortical electrogenesis and poorly-structured epileptiform activity in the occipital-parietal-posterior temporal regions.

**Fig. 1:**
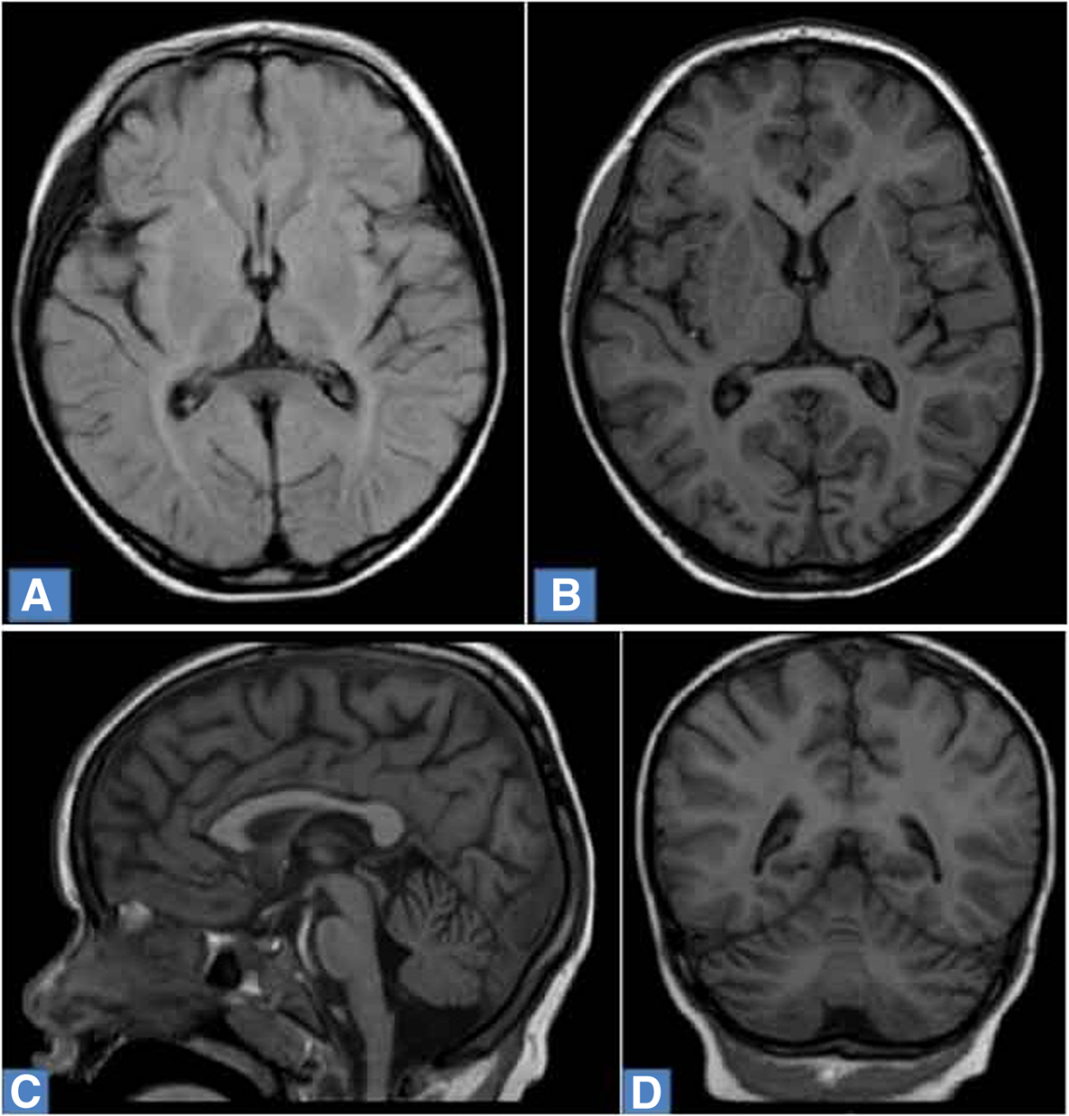
Brain MRI of 5-year-old girl with NCL7. **a**. Diffuse cortical atrophy, increased intensity of the MR signal in periventricular white matter. Axial T2 weighted FLAIR image. **b**. Diffuse cortical and subcortical atrophy of the brain. Axial T1 weighted image. **c**. Atrophy of the cerebellum, cortical atrophy of the brain. Sagittal T1 weighted image. **d**. Diffuse cortical and subcortical atrophy of the brain, atrophy of the cerebellum. Frontal T1 weighted image

MRI (Magnetic Resonance Imaging) revealed cortical atrophy, periventricular leukopathy of both hemispheres of the brain and atrophy of the cerebellum (Fig. 1).

ECG (electrocardiography) showed severe sinus bradyarrhythmia. The heart rate was 48–84 bpm.

In the hospital, she received treatment with anticonvulsant drugs: topiramate (100 mg/day) and levetiracetam (1200 mg/day).

Clinical exome sequencing was carried out by Genotek Ltd. Genomic DNA from peripheral blood sample was extracted using QIAamp DNA Mini Kit (Qiagen) according to manufacturerʼs protocol. DNA libraries were prepared using AmpliSeq Exome (ThermoFisher Scientific) according to manufacturerʼs protocol. Sequencing was performed on Ion Proton System (ThermoFisher Scientific). After sequencing we trimmed 3′-nucleotides with read quality below 10 using Cutadapt [19]. Raw reads were aligned to reference genome hg19 (GRCh37.p13) using BWA MEM [20]. FastQС was used for data quality control [21]. We called short variants using GATK HaplotypeCaller [22] according to GATK Best Practices DNA-seq [23, 24]. The effect of each mutation was assessed using snpEff [25] To assess pathogenicity and conservatism, the data was extracted from the dbNSFP [26], Clinvar [27, 28], OMIM database (Online Mendelian Inheritance in Man) [29] and HGMD [30], as well as using the SIFT [31] and PolyPhen-2 [32, 33] utilities to predict pathogenicity of the mutation. Information on the frequency of mutations was taken from 1000Genomes project [34, 35], ExAC [36, 37] and Genotek frequency data. Description of mutations and their pathogenicity were predicted according to the Standards and Guidelines developed by ACMG (American College of Medical Genetics and Genomics), AMP (Association for Molecular Pathology) and CAP (College of American Pathologists) [38]. Copy number alterations were determined using CNVkit [39].

*MFSD8* variant identified by exome sequencing was confirmed by Sanger sequencing.

## Discussion and conclusions

In this article we described a case of 5-year old girl with motor and mental deterioration, progressive vision loss, stereotypies, action myoclonus and epilepsy. Disease had Rett-like onset (psychomotor regression, stereotypic hands movements). Therefore, prior to clinical exome sequencing Rett syndrome was excluded by analysis of *MeCP2.* Also analysis of frequent mutations and biochemical indices was performed for several diseases: aminoacidopathies, organic aciduria, NCL1, NCL2, mitochondrial fatty acid betta-oxidation disorders, MELAS (mitochondrial encephalopathy, lactic acidosis and stroke-like episodes), MERRF syndrome (myoclonic epilepsy with ragged red fibers), NARP (neuropathy, ataxia, and retinitis pigmentosa). All results were negative.

**Fig. 2:**
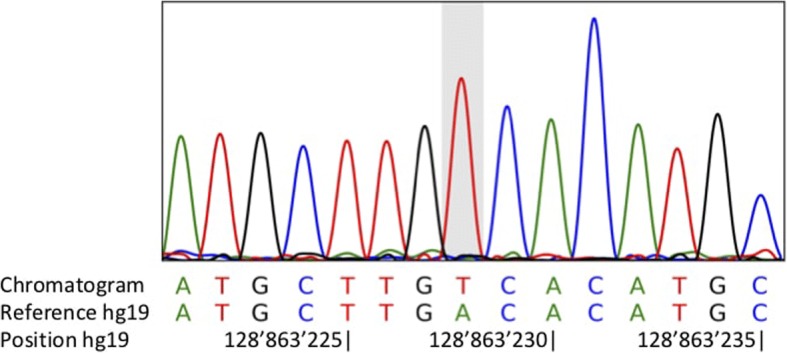
Electrophoregram from Sanger sequencing of the proband showing the homozygous c.525 T > A change in exon 6 of *MFSD8*, which predicts a p.Cys175Ter change

Exome sequencing revealed homozygous c.525 T > A variant in exon 6 of the *MFSD8* (NM_152778.2). This variant leads to a premature stop codon (p.Cys175Ter). This homozygous mutation was confirmed by Sаnger sequencing (Fig. 2).

This mutation is not reported in 60,706 subjects in ExAC [34] or in 2535 subjects in 1000 Genomes Browser [32]. This mutation was not found in our 2000 in-house exomes.

Discovered variant was predicted to be pathogenic. This variant affects 175 aa of protein in transmembrane ɑ helix. This nonsense variant may result in truncated protein that is nonfunctional or leads to degradation of mRNA through nonsense-mediated decay [40].

This mutation was not described previously, but homozygous or compound heterozygous mutations in this gene are associated with ceroid lipofuscinosis. To date, 38 mutations in *MFSD8* were described previously, most being homozygous missense mutations [5, 11]. This mutations predominantly lead to NCL7 disease - subtype of vLINCL form. Phenotypes of almost all affected individuals are very similar regardless of mutation type [41].

The symptoms of NCL7 disease typically begin between ages 2 and 11 (mean onset 5 years). The initial features usually include seizures and the loss of previously acquired skills. As the disease progressed, mental regression, myoclonus, speech impairment, loss of vision developed [15].

*MFSD8* gene, which is located on chromosome 4q28.1-q28.2, encodes CLN7, a putative lysosomal transporter with suggested topology of 12 transmembrane domains that was shown to be localized to the lysosomal membrane and belongs to the major facilitator superfamily (MFS). These proteins are single-polypeptide carriers that are able to transport small solutes by using chemiosmotic ion gradients [42]. Specific molecules that MFSD8 transports across the lysosomal membrane have not been identified. Although this protein is ubiquitously expressed, high transcript concentrations have been identified in several brain locations, such as cerebellar cortex and hippocampus [43].

Despite advances in diagnosis of neurodegenerative disorders, NCLs remain a challenge for pediatric neurologists, because clinical signs in young children or toddlers are subtle and often overlap with other congenital neurodegenerative diseases, such as mitochondrial disorders, Rett syndrome or early-onset Parkinsonism. Craiu with colleagues concluded that NCL should be suspected in patients with Rett-like phenotype at onset and negative *MECP2* mutation [15]. Disease of our patient also had Rett-like signs at onset which caused diagnostic delay. Both Rett syndrome and NCLs usually have normal development until age 9–24 months. Patient in Craiu et al. article has NCL7 disease with Rett-like onset at 18 months. Our case has late manifestation at 2,5 years which made it more difficult to diagnose. Increase in genetic understanding of NCLs has led to improved diagnostic approaches. Our study revealed that early ophthalmological examination of patients with motor and mental regression can be useful for diagnosis.

Although there is no treatment for this condition, correct and early diagnosis is important for appropriate low-vision management, educational planning, and genetic counseling.

This report describes the first case of NCL7 disease in Russia. Our findings expanded variant diversity of *MFSD8* and proved value of exome sequencing for pediatric NCLs.

## Abbreviations

ACMG: American College of Medical Genetics and Genomics
ECG: Electrocardiography
EEG: Electroencephalography
MRI: Magnetic Resonance Imaging
NCL: Neuronal Ceroid Lipofuscinosis
NGS: Next Generation Sequencing
